# GTP hydrolysis by *Synechocystis* IM30 does not decisively affect its membrane remodeling activity

**DOI:** 10.1038/s41598-020-66818-9

**Published:** 2020-06-17

**Authors:** Benedikt Junglas, Carmen Siebenaller, Lukas Schlösser, Nadja Hellmann, Dirk Schneider

**Affiliations:** Department of Chemistry, Biochemistry, Johannes Gutenberg University Mainz, 55128 Mainz, Germany

**Keywords:** Bioenergetics, Membrane biophysics, Membrane structure and assembly, Chloroplasts, Chloroplasts, Bacteria, Bacterial development

## Abstract

The function of IM30 (also known as Vipp1) is linked to protection and/or remodeling of the thylakoid membrane system in chloroplasts and cyanobacteria. Recently, it has been revealed that the *Arabidopsis* IM30 protein exhibits GTP hydrolyzing activity *in vitro*, which was unexpected, as IM30 does not show any classical GTPase features. In the present study, we addressed the question, whether an apparent GTPase activity is conserved in IM30 proteins and can also be observed for IM30 of the cyanobacterium *Synechocystis* sp. PCC 6803. We show that *Synechocystis* IM30 is indeed able to bind and hydrolyze GTP followed by the release of P_i_. Yet, the apparent GTPase activity of *Synechocystis* IM30 does not depend on Mg^2+^, which, together with the lack of classical GTPase features, renders IM30 an atypical GTPase. To elucidate the impact of this cryptic GTPase activity on the membrane remodeling activity of IM30, we tested whether GTP hydrolysis influences IM30 membrane binding and/or IM30-mediated membrane fusion. We show that membrane remodeling by *Synechocystis* IM30 is slightly affected by nucleotides. Yet, despite IM30 clearly catalyzing GTP hydrolysis, this does not seem to be vital for its membrane remodeling function.

## Introduction

IM30, the inner membrane-associated protein of 30 kDa (also known as Vipp1 (vesicle-inducing protein in plastids 1)), is present in cyanobacteria as well as in chloroplasts of higher plants and algae^1^. In chloroplasts, the protein has a triple localization: It can be found soluble in the stroma as well as bound to the inner chloroplast envelope membrane and the thylakoid membrane (TM)^2^. Similarly, IM30 is also found in a soluble form as well as bound to the cytoplasmic membrane and TMs of cyanobacteria^3^.

IM30 is a predominantly α-helical protein predicted to consist of seven α-helices, which are able to form coiled-coil structures^3–5^. *In vitro*, IM30 monomers assemble to form diverse higher ordered homo-oligomers with molecular masses exceeding 2 MDa^4–7^. *In vivo*, in cyanobacteria and in chloroplasts, GFP-labeled IM30 forms large dynamic assemblies, which are mainly located at TM regions with high membrane curvature^8–10^.

While diverse functions have been attributed to IM30 in the past^1,2,10–24^, most observations clearly indicate that the protein is membrane-active and involved in the biogenesis, dynamics and/or stabilization of internal membranes in chloroplasts and cyanobacteria (recently reviewed in^25^). Thus, the physiological function of IM30 appears to be linked to dynamic membrane remodeling^25–27^, likely involving membrane fusion/fission events, which have been observed in plant chloroplasts^28–31^. In fact, dynamic rearrangement of the TM system is crucial for adaptation of photosynthetic processes to altering environmental conditions, most importantly in response to altering light intensities^32,33^. While TM dynamics is studied to some extent in chloroplasts, direct observations of TM dynamics in cyanobacteria are still limited, although TMs in cyanobacteria are assumed to be as dynamic as in chloroplasts^34,35^. Nevertheless, a machinery mediating such membrane remodeling processes has not been unambiguously identified yet, neither in chloroplasts nor in cyanobacteria^36^, and IM30 is currently the only protein in chloroplast and cyanobacteria recognized to have a membrane fusion activity^23^. Importantly, light not only triggers rearrangement of the TM system but also rearrangement and redistribution of intracellular IM30 clusters at internal membranes^8,26^, which again indicates a connection between TM dynamics and IM30 oligomer formation^25^.

In several aspects, the membrane activity of IM30 resembles other membrane-active proteins, as IM30 is involved in membrane fusion, protection and/or stabilization^10,15,16,18,23,37,38^ and has a strong tendency to oligomerize and to form higher ordered structures^3,4,7^. Yet, in most thus far described systems, membrane remodeling, especially membrane fission and fusion, are typically connected to nucleotide hydrolysis. Thus, it was somewhat intriguing to suspect a nucleotide binding/hydrolysis function also for IM30, a protein that can mediate membrane fusion^23^. Consistent with this idea, Ohnishi *et al*. recently suggested that recombinant *Arabidopsis* IM30 (*Ara*IM30) has an intrinsic GTPase activity despite lacking classical features of GTPases, implying that IM30 belongs to a new class of membrane-remodeling GTPases^39^. Yet, the molecular details of the IM30 membrane activity have been extensively studied *in vitro* in recent years, and all membrane-related activities were already observed in the absence of nucleotides. Thus, the question arises whether GTP binding and/or hydrolysis have any impact on the structure and/or activity of IM30.

In the present study, we have analyzed the apparent GTPase activity of IM30 of the cyanobacterium *Synechocystis* sp. PCC 6803. We have tested the influence of nucleotides on IM30-triggered membrane fusion and lipid organization *in vitro*. Our results suggest that the *Synechocystis* IM30 also has a low GTP hydrolyzing activity, as observed before with the *Arabidopsis* homolog, yet nucleotides have only minor effects on the *in vitro* membrane remodeling activity of IM30, and therefore nucleotide binding/hydrolysis does not appear to critically affect IM30-triggered membrane remodeling.

## Results

### SynIM30 has an apparent GTPase activity

The *Arabidopsis* IM30 protein (*Ara*IM30) appears to have a GTPase activity under defined *in vitro* conditions^39^. As this has never been described for any IM30 protein before, we first aimed to elucidate whether this apparent activity is conserved in IM30 proteins. Therefore, we analyzed the interaction of a cyanobacterial IM30 homolog (*Synechocystis* IM30; *Syn*IM30) with GTP.

Initially, we tested whether *Syn*IM30 hydrolyzes GTP, using the malachite green-based assay that has also been used by Ohnishi *et al*. to determine the apparent GTPase activity of *Ara*IM30^39^.

**Figure 1:**
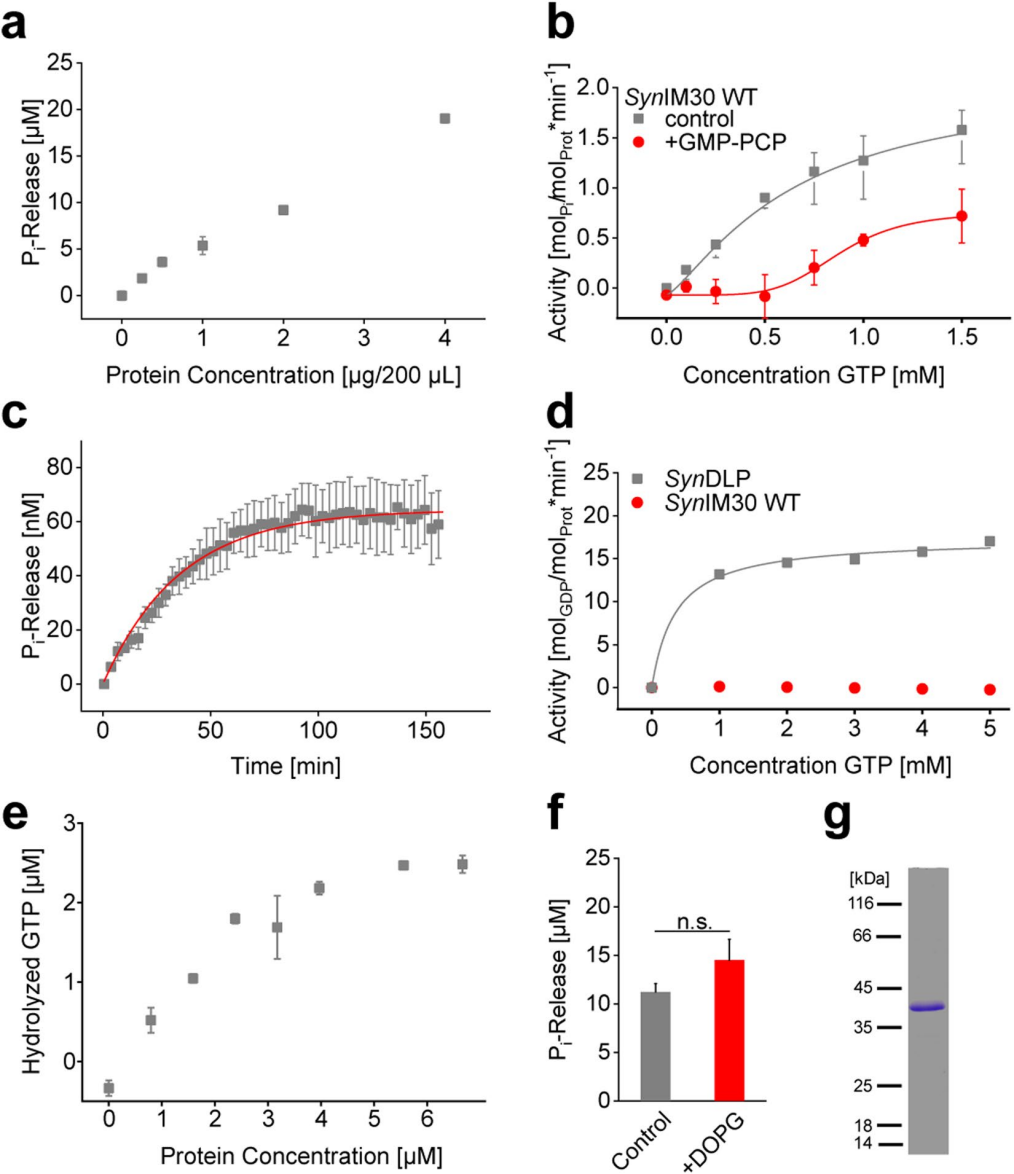
IM30 GTPase activity was characterized by multiple assays. (**a**) A malachite green-based assay was used to measure the P_i_ release catalyzed by *Syn*IM30 WT (0.5 mM GTP; 2.5 mM Mg^2+^; 30 min at 37 °C). (Error bars represent SD, n = 3). (**b**) The enzymatic properties of the GTP hydrolysis caused by *Syn*IM30 were analyzed with a malachite green-based assay in absence and presence of 0.5 mM GMP-PCP (0.1 µM IM30 WT; 2.5 mM Mg^2+^; 30 min at 37 °C). Fitting the data with a Hill equation derived model yielded ${V}_{max}$ value of 2.16 ± 0.33 mol GTP per mol IM30 per min, *K*_*m*_ = 0.65 ± 0.14 mM; *n* = 1.29 ± 0.09 and ${V}_{max}$ = 0.83 ± 0.33 min^−1^; *K*_*m*_ = 0.88 ± 0.16 mM; *n* = 5.19 ± 3.64 in presence of GMP-PCP. (R^2^ = 0.99835 and R^2^ = 0.95888; SD, n = 3). (**c**) A fluorescence-based continuous test for P_i_ release was used to determine *Syn*IM30 GTPase activity in real-time (0.1 µM IM30 WT; 10 µM GTP; 2.5 mM Mg^2+^; 37 °C). Fitting the data with a mono-exponential function yielded *v*_*0*_ = 1.79 ± 0.02 min^−1^ and $\eta$ = 2.79 ± 0.05 *10^−2^ (R^2^ = 0.97898; SD, n = 3). (**d**) A PK/LDH-coupled assay was used to determine the GTPase activity independent of the P_i_ release. *Syn*IM30 WT did not show significant activity (SD, n = 3). As a control, a GTPase with known activity was used (*Syn*DLP; n = 1). (**e)** A luciferase-coupled GTPase assay was used to measure the amount of hydrolyzed GTP over 120 min at 37 °C in the presence of increasing *Syn*IM30 concentrations (4 µM GTP). (Error bars represent SD, n = 3) (**f**) GTPase activity of IM30 WT was measured by the malachite green-based assay in absence and presence of 30 µM DOPG (2 µg/200 µL protein, 0.5 mM GTP; 2.5 mM Mg^2+^; 30 min at 37 °C). The GTPase activity is not significantly increased in the presence of the liposomes. (SD, n = 3, two-sample Student´s t-test, significance level p > 0.05). (**g**) The purity of *Syn*IM30 WT heterologously expressed in *E. coli* and purified via Ni^2+^-affinity chromatography was analyzed by SDS-PAGE.

Indeed, we observed protein-dependent P_i_ production, *i.e*. GTP hydrolysis, in perfect agreement with the findings of Ohnishi *et al*., reaching about 20 µM P_i_ release at a protein concentration of 2 µg/100 µL (Fig. 1a). To rule out that P_i_ release of the protein originated from contamination with a GTPase, we analyzed the purity of the protein via SDS-PAGE, whereby no other protein was identified besides IM30 (Fig. 1g). Although this does not finally exclude any contamination with a highly active GTPase, the missing GTPase activity of an IM30 mutant (Fig. 3a), purified following the exact same protocol, is a strong hint that the observed GTPase activity can indeed be exclusively ascribed to IM30, as further discussed below.

Subsequently, we used the malachite green-based assay to further characterize the apparent GTPase activity of IM30. The steady-state activity of IM30 in dependence on the GTP concentration could be described by a slightly sigmoidal curve (Fig. 1b). Fitting the experimental data with the Hill-equation (Eq. 2) resulted in a ${k}_{cat}$ value of 2.16 ± 0.33 mol GTP per mol IM30 per min, a Michaelis-Menten constant of 0.65 ± 0.14 mM and a Hill coefficient of 1.29 ± 0.09. Addition of the non-hydrolyzable GTP analog GMP-PCP, which is expected to act as a competitive inhibitor, strongly increased the cooperativity, as evidenced by the increased Hill coefficient (n = 5.19 ± 3.64) (Fig. 1b).

As the malachite green-based assay is not capable of measuring reaction kinetics, we next applied a continuous assay for P_i_ release, which is based on a recombinant *E. coli* phosphate-binding protein labeled with the fluorophore MDCC^40^. This assay indeed allowed monitoring the kinetics of the apparent IM30 GTPase activity, measured as the P_i_ release (Fig. 1c). Fitting the data with a model for non-linear, steady-state enzyme kinetics and assuming a first-order process^41^ yielded a *k*_*cat*_ (Eqs. 3 and 4) in presence of 10 µM GTP (1.79 ± 0.02 * 10^−2^ min^−1^) (Fig. 1c). However, these measurements could be performed solely at very low GTP concentrations, as the P_i_ sensor has an extremely high sensitivity for P_i_.

Although specifically established for measuring NTPase activities, the malachite green-based assay is prone to artifacts, and *e.g*. protein binding to the malachite complex or acids can induce NTP hydrolysis, causing false positive results^42^. Thus, we aimed to apply an established PK/LDH-coupled assay that measured GTP hydrolysis by coupling the regeneration of GTP from GDP to a decline in NADH concentration, which can be monitored by absorbance at 340 nm^43^. Surprisingly, when using this assay we were not able to measure a significant amount of GTP hydrolysis (Fig. 1d). Even doubling the *Syn*IM30 concentration did not produce a detectable signal (data not shown). Based on the P_i_ release determined with the malachite green assay (20 µM P_i_/30 min) we calculated the expected change in absorption at 340 nm, amounting to ΔOD = 0.124 in 30 min. This should be easily detectable if GDP is released. To ensure that the assay works properly, we also analyzed the GTPase activity of *Syn*DLP, a canonical P-loop GTPase of the cyanobacterium *Synechocystis* sp. PCC6803^36^, as a positive control (Fig. 1d).

Since we were not able to detect any GTP hydrolysis using the PK/LDH-coupled assay, we wondered whether the observed apparent GTPase activity might be caused by P_i_ leaking into the reaction mixture, *e.g*. by release of P_i_ bound to IM30 rather than by GTP hydrolysis. To test this, we used a luciferase-coupled GTPase assay, which directly depends on the GTP concentration rather than P_i_-release. In this assay, non-hydrolyzed GTP is enzymatically converted to ATP and the resulting ATP concentration is subsequently measured in the luciferase reaction. Here we observed a steady decrease of the GTP concentration in the presence of IM30 in a protein concentration-dependent manner, as expected for a GTP hydrolyzing activity (Fig. 1e). Thus, we conclude that *Syn*IM30 can indeed catalyze the hydrolysis of GTP.

The activity of many membrane-active GTPases is modulated when the protein interacts with lipid membranes, and interaction of *e.g*. dynamins with membrane surfaces can increase the GTPase activity by a factor of 40^44,45^. As IM30 binds to negatively charged PG liposome surfaces^23,24,37,46^, we next assayed GTP hydrolysis by *Syn*IM30 in absence *vs*. in presence of DOPG liposomes. While our measurements were limited to low lipid concentrations, as the liposomes created a strong background signal in the assay, we did not observe a significant increase in GTP hydrolysis in presence of DOPG liposomes (p = 0.066) (Fig. 1f).

In summary, *Syn*IM30 clearly has an apparent GTPase activity *in vitro*, as observed before with the *Ara*IM30 protein. This activity can be modulated by a classical GTPase inhibitor and does not seem to be affected by membranes.

### IM30 binds and hydrolyzes GTP in the presence and absence of Mg^2+^

Thus far, apparent GTP hydrolysis was always tested in presence of Mg^2+^, as the activity of NTPases typically requires the presence of Mg^2+^. Notably, the interplay of IM30, Mg^2+^ and GTP is of special interest, as also the membrane fusion activity of IM30 depends on Mg^2+^ ^23^, and binding of Mg^2+^ to IM30 even causes rearrangement of the IM30 structure^47^.

**Figure 2:**
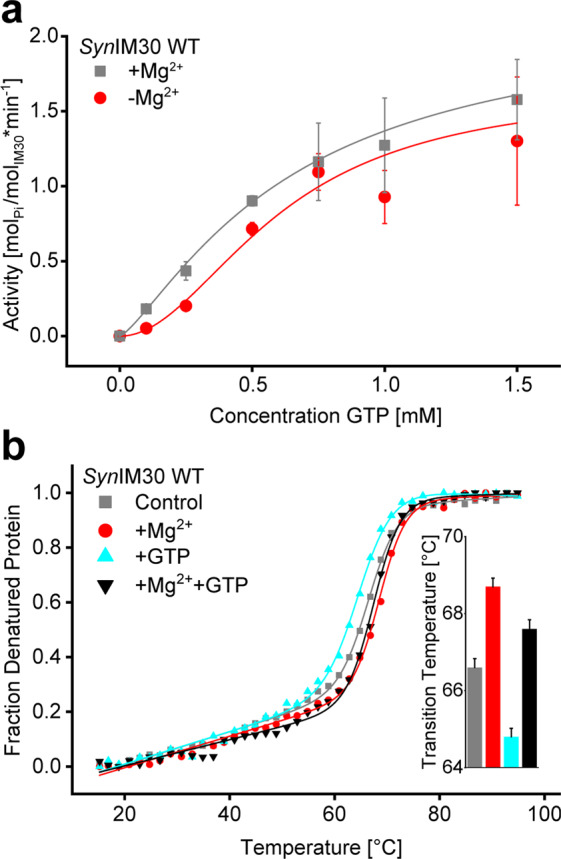
GTP binds to IM30 in presence and absence of Mg^2+^. (**a**) GTP hydrolysis caused by IM30 WT in absence of Mg^2+^ was analyzed using a malachite green-based assay (0.1 µM IM30 WT; 0 mM Mg^2+^; 30 min at 37 °C). Fitting of the data with a Hill equation derived model yielded ${V}_{max}$ = 1.66 ± 0.55 min^−1^; *K*_*m*_ = 0.61 ± 0.20 mM; *n* = 2.00 ± 0.27 in absence of Mg^2+^. (R^2^ = 0.96951; SD, n = 3). Data in presence of Mg^2+^ were taken from Fig. 1b (control). (**b**) Thermal denaturation of IM30 WT in the absence and presence of GTP and Mg^2+^ was monitored by CD-spectroscopy at 222 nm. The raw signal was converted to the fraction of denatured protein (*f*_*D*_). Fitting the data with an adapted Boltzmann-fit yielded: *T*_*m*_ = 66.6 ± 0.2 °C (0 mM GTP; 0 mM Mg^2+^) (R^2^ = 0.99866); *T*_*m*_ = 68.7 ± 0.2 °C (0 mM GTP; 2.5 mM Mg^2+^) (R^2^ = 0.99841); *T*_*m*_ = 64.8 ± 0.2 °C (0.5 mM GTP; 0 mM Mg^2+^) (R^2^ = 0.99836) and *T*_*m*_ = 67.5 ± 0.2 °C (0.5 mM GTP; 2.5 mM Mg^2+^) (R^2^ = 0.99784). Error bars represent errors from the fitting of the data.

To test the Mg^2+^ dependence of the IM30 GTPase activity we determined GTP hydrolysis rates in the absence of Mg^2+^. Surprisingly, the *K*_*M*_ value (0.61 ± 0.20 mM) determined for GTP hydrolysis was essentially identical, regardless of the presence of Mg^2+^. Similarly, the ${v}_{max}$ value (1.65 ± 0.55 min^−1^) did not significantly differ from the value determined in the presence of Mg^2+^ (2.16 ± 0.33 min^−1^) (Fig. 2a). Thus, Mg^2+^ does obviously not have a strong impact on GTP hydrolysis by *Syn*IM30. Yet, cooperativity was strongly increased in absence of Mg^2+^, reaching a value of 2.00 ± 0.27, which nicely supports the recently described Mg^2+^-induced change of the IM30 structure and stability^47^. In fact, addition of Mg^2+^ *e.g*. increased the thermal stability of IM30 as indicated by *T*_*m*_ values of 66.6 ± 0.2 °C and 68.7 ± 0.2 °C in absence and presence of 2.5 mM Mg^2+^, respectively (Fig. 2b). However, an increased stability of *Syn*IM30 was observed when 0.5 mM GTP and 2.5 mM Mg^2+^ were present together (*T*_*m*_ 67.6 ± 0.2 °C) compared to *Syn*IM30 in presence of solely 2.5 mM GTP, where the *T*_*m*_ was as low as 64.6 ± 0.2 °C (Fig. 2b). This suggests that GTP binding affects the structure and stability of IM30 to some extent, and Mg^2+^ and GTP do not operate synergistically but have opposing effects on the *Syn*IM30 stability.

Thus, GTP binding to *Syn*IM30 and *Syn*IM30-mediated GTP hydrolysis appear to be independent of Mg^2+^, which is rather uncommon for GTPases.

### GTP hydrolysis depends on the oligomeric state of IM30

**Figure 3:**
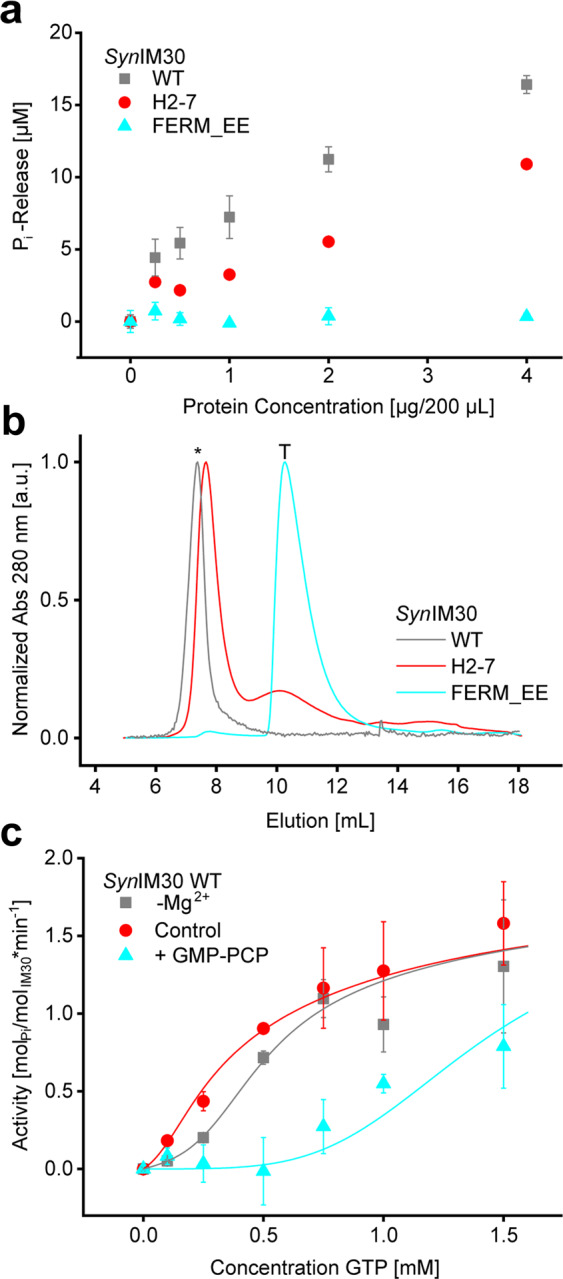
The oligomeric state of IM30 is crucial for GTP hydrolysis. (**a**) The malachite green-based assay was used to compare the GTPase activity of IM30 variants with different oligomeric states (0.5 mM GTP; 2.5 mM Mg^2+^; 30 min at 37 °C). Compared to IM30 WT, H2-7 has a reduced GTPase activity. The tetrameric IM30 FERM_EE has no detectable GTPase activity. (Error bars represent SD, n = 3). (**b**) The oligomeric state of IM30 variants was determined by SEC (Superose 12 10/300 GL column). The elution volumes determined are 7.4 mL for IM30 WT (>300 kDa); 7.7 mL (>300 kDa) and 10.2 mL (105 kDa) for IM30 H2-7 and 10.3 mL (100 kDa) for IM30 FERM_EE. The asterisk marks the position of the void volume. T marks the position of an IM30 tetramer. (**c**) The data of the malachite green assay of IM30 WT in absence and presence of Mg^2+^ and after addition of GMP-PCP were analyzed using a more sophisticated model (described in detail in the Supplemental Data 3), to disentangle the mechanism of GTP-hydrolysis. Error bars represent SD, n = 3. Fit: n = 12, allosteric model (see Supplemental Data 3).

Based on studies using truncated *Ara*IM30 it has been suggested that helix 1 is crucial for GTP binding to *Ara*IM30 and for its apparent GTPase activity^39^. As we assume that the apparent GTPase activity is conserved in IM30 proteins of different origins, we next tested the GTP hydrolyzing activity of the recently described *Syn*IM30 variant H2-7, where helix 1 was deleted^46^. Although this mutant showed a decreased P_i_ release rate compared to the wt protein (Fig. 3a), P_i_ release was decreased only by about 40% (*i.e*. to roughly 60% of the wt levels), whereas the GTPase activity of the *Ara*IM30 protein was completely abolished when helix 1 was deleted^39^. However, the *Ara*IM30 protein does not form high molecular mass oligomers anymore when helix 1 is deleted^4,39^, whereas IM30 of *Chlamydomonas rheinhardtii*^5^ and the here analyzed *Syn*IM30 do^46^, as recently discussed in^25^. Yet, a fraction of smaller oligomers was also identified in SEC analyses of the *Syn*IM30 H2-7 protein (see Fig. 3b). Thus, the disturbed formation of high molecular mass oligomers might explain the abolished GTPase activity of the *Ara*IM30 H2-7 protein^39^, and the reduced amount of released P_i_ by *Syn*IM30 H2-7 (Fig. 3b).

To further test the hypothesis that the decreased oligomeric state, and not deletion of helix 1, has caused the reduced apparent GTPase activity, we next generated a *Syn*IM30 variant that exclusively forms small oligomers (mainly tetramers in buffers with low ionic strength) (*Syn*IM30 FERM_EE) and no high molecular mass oligomers (Fig. 3b). This mutant is based on the *Syn*IM30_FERM variant described recently^7,37^, but additionally E83 and E84 in the loop region of helix 2 and 3 were mutated to overcome the problem of unspecific aggregation upon prolonged exposure to HEPES buffer. In line with our hypothesis, this variant had no detectable GTPase activity (Fig. 3a). Note that also *Syn*IM30_FERM does not exhibit detectable GTPase activity (Suppl. Fig. 1).

Thus, we conclude that the IM30 GTPase activity depends on the IM30 oligomeric state, and formation of high molecular mass oligomers is a prerequisite of GTP hydrolysis. A lack of high molecular mass oligomers seems to be the major factor for GTP hydrolysis since helix 1 is unchanged in the tetrameric *Syn*IM30 variant. Together, these results clearly suggest that not helix 1 but the formation of high molecular mass oligomers is crucial for GTP hydrolysis. In addition, the absent GTP hydrolyzing activity of the mutant protein confirms that the GTPase activity observed with the IM30 wt (Fig. 1) was not caused by an (unintended) co-purification of an (unknown) GTPase.

Considering that IM30 forms oligomers, the sigmoidal shape of the GTPase activity in presence of GMP-PCP (Fig. 1b) and in absence of Mg^2+^ (Fig. 2a) might well reflect the interaction of GTP-binding sites within the protein. Indeed, the data describing the GTPase activity of IM30 can be well depicted by a cooperative two-state model (Fig. 3c, see Suppl. Fig. 3 for details). The increase in cooperativity in presence of GMP-PCP can be explained by competitive binding, which would shift the distribution towards the low-affinity state, requiring more GTP to yield measurable activity. Formally, also binding to an allosteric site could explain the data. Both variants of the two-state model describe the data similarly well (see Supplemental Data 3). The number of binding sites interacting, giving rise to the observed cooperativity, is ≥8 (Suppl. Fig. 3d). Thus, these data correspond well with the idea of having multiple subunits interacting with each other during GTP binding/hydrolysis, which explains the necessity of IM30 rings/high molecular weight complexes for GTPase activity.

### IM30 binding to membrane surfaces is not influenced by GTP

IM30 binds to negatively charged membrane surfaces and is involved in membrane remodeling^16,23,37^. Thus, we next studied the impact of GTP on binding of *Syn*IM30 to negatively charged membrane surfaces.

In the presence of Mg^2+^, *Syn*IM30 exhibits membrane fusion activity, and such membrane fusion events can disturb other assays, involving IM30 and liposomes^23^. Luckily, GTP binding to *Syn*IM30 and GTP hydrolysis were observed also in absence of Mg^2+^ (Fig. 2a,b). Thus, we were able to analyze the influence of nucleotide binding to *Syn*IM30 on membrane binding independent of the effect on IM30-mediated membrane fusion.

**Figure 4:**
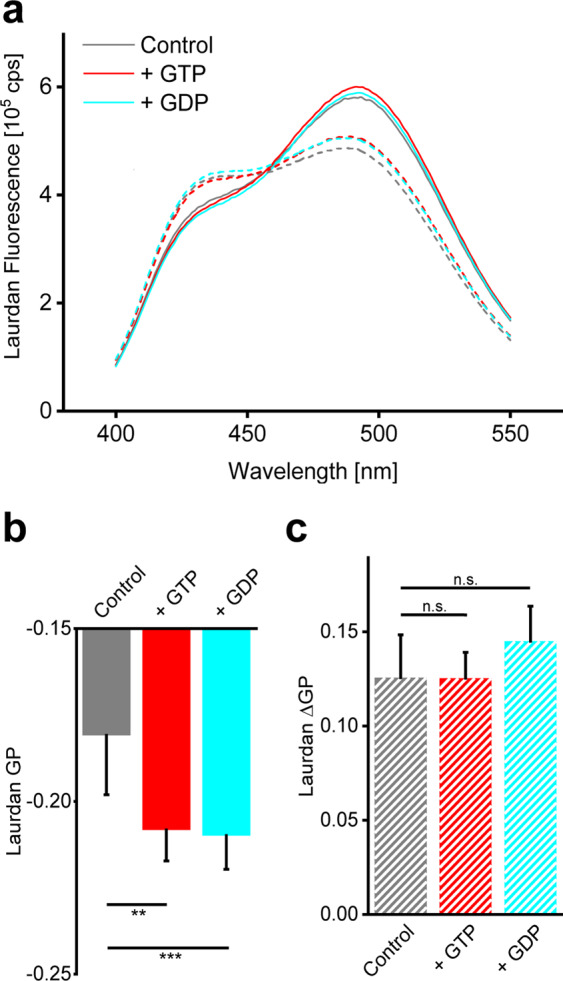
The presence of the nucleotides GTP and GDP does not affect the IM30-mediated change of the DOPG lipid order. (**a**) Laurdan fluorescence emission spectra of 0.1 mM DOPG liposomes were recorded in absence and presence of 2.5 mM GTP or GDP and 1 µM *Syn*IM30 WT. Solid lines show the spectra in absence of IM30. Dashed lines show the spectra in the presence of *Syn*IM30 WT. Binding of IM30 leads to a clear shift of the Laurdan fluorescence maximum. (**b**) The GP value was calculated for DOPG liposomes in the presence and absence of GTP and GDP without addition of *Syn*IM30. Addition of the nucleotides leads to a small, but still significant decrease of the GP-value. SD, n = 6 two-sample Student´s t-test, *p < 0.05, **p < 0.01, ***p < 0.005. **(c**) The ΔGP after addition of *Syn*IM30 WT to DOPG liposomes is not significantly changed in the presence of the nucleotides GTP or GDP, respectively. (SD, n = 6 two-sample Student´s t-test).

First, we analyzed whether GTP has an impact on the propensity of *Syn*IM30 to interact with negatively charged DOPG liposomes. It has previously been shown that binding of *Syn*IM30 to DOPG membranes can be monitored via steady-state Laurdan fluorescence spectroscopy^23,37^. As shown in Fig. 4a,b, the Laurdan fluorescence emission spectrum was already altered upon addition of solely GTP or GDP, even when *Syn*IM30 was not present, albeit only to a minor extent. Thus, the nucleotides appear to interact with the liposomes, resulting in a minor change of the Laurdan fluorescence emission. However, when *Syn*IM30 was added to the liposomes, the Laurdan fluorescence spectrum changed far more significantly, and an altered fluorescence spectrum was observed in absence as well as in presence of GTP or GDP. All spectra recorded in presence of *Syn*IM30 (Fig. 4a) showed an increased fluorescence emission at 440 nm and a decreased emission at 490 nm, leading to an increase of the GP-value. This indicates a decreased polarity of Laurdan’s environment, as occurring when the lipid order increases. The changes in the lipid order, represented by the ΔGP values, showed no significant impact of GTP or GDP on the interaction of *Syn*IM30 with DOPG liposomes (Fig. 4c), indicating that GTP binding and/or hydrolysis by *Syn*IM30 do not affect binding of IM30 to lipid bilayers, at least not in the absence of Mg^2+^. Unfortunately, it was not possible to analyze *Syn*IM30 binding to DOPG liposomes in the presence of Mg^2+^ due to direct binding of Mg^2+^ to DOPG membranes as well as to to *Syn*IM30^47^ and side-effects caused by *Syn*IM30-mediated membrane fusion^23^.

### GTP and GDP do only marginally affect the kinetics of IM30-mediated membrane fusion

*Syn*IM30 has a membrane fusion activity when Mg^2+^ is present (Hennig *et al*., 2015). Consequently, we next analyzed the potential impact of GTP and GDP on IM30-induced liposome fusion in the presence of Mg^2+^.

Membrane fusion was measured using a FRET-based liposome fusion assay, as described in detail previously^23^. Importantly, in presence of the nucleotides, the concentration of MgCl_2_ had to be adjusted, as GTP and GDP coordinate Mg^2+^ via their phosphate groups^48^ and thereby lower the effective concentration of “free” Mg^2+^ which clearly influences the membrane fusion process^23^. The concentration of additionally “needed” MgCl_2_ was determined experimentally in the presence of GTP and GDP, as described in the “Experimental Procedures” section. Note that controls containing exclusively IM30, Mg^2+^, the nucleotides or the respective Mg^2^/nucleotide mixture did not show any membrane fusion activity (data not shown).

**Figure 5:**
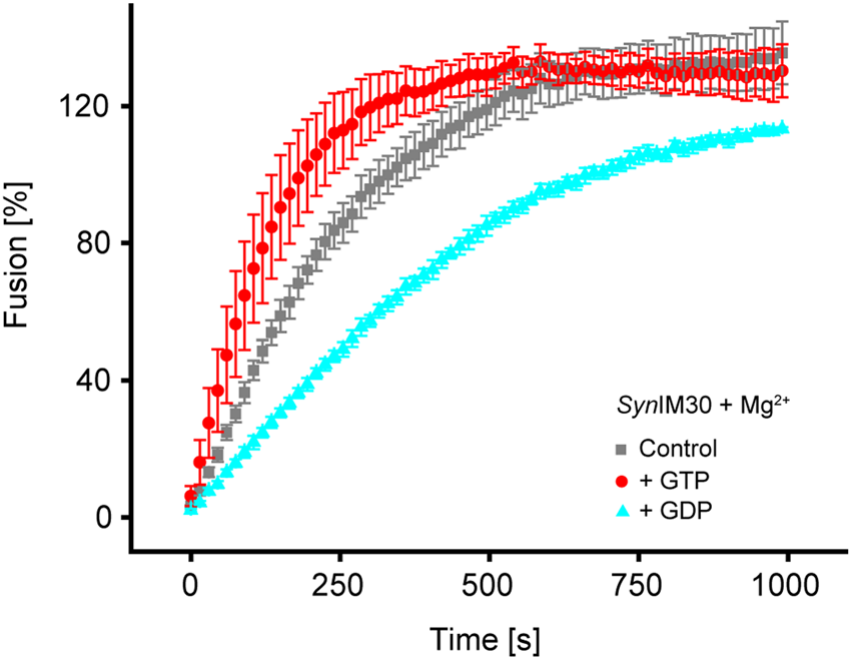
IM30-mediated membrane fusion is modulated to some extent by GTP and GDP. Liposome fusion was measured in the presence of 1 µM *Syn*IM30 WT, 2.5 mM nucleotide and 6 mM Mg^2+^. Because of binding of free Mg^2+^ by the nucleotides, “extra” needed Mg^2+^, needed to obtain the same membrane destabilization, was also added (2.5 mM for GDP, 4.25 mM for GTP). The presence of GTP leads to a slight increase in the fusion rate, whereas the presence of GDP leads to a slower fusion process. (SD, n = 3).

In contrast to membrane fusion observed in absence of nucleotides, the fusion assay indicated a slightly increased fusion rate in presence of GTP, while slightly less liposome fusion was observed in presence of GDP (Fig. 5). The initial fusion rate increased from 0.58 ± 0.20%/s in the absence of nucleotides to 0.80 ± 0.22%/s in the presence of GTP. In contrast, addition of GDP lowered the initial fusion rate to 0.33 ± 0.04%/s. However, in all cases the fusion curves appear to level out at approximately the same amount of total fusion.

Clearly, GTP binding/hydrolysis is not unequivocally required for IM30-mediated membrane fusion, and the presence of GTP or GDP does neither abolish nor exclusively trigger membrane fusion. We conclude therefore that the GTP hydrolyzing activity of IM30 is not a key regulator of the membrane fusion process, as it is not switching the IM30 membrane fusion activity from a completely active to an inactive state, or *vice versa*. Yet, as we did observe small effects on the fusion kinetics in the presence of GTP and GDP, we cannot completely rule out the possibility that nucleotide binding and/or GTP hydrolysis modulate IM30-mediated membrane fusion.

## Discussion

### SynIM30 has a low GTP binding affinity and hydrolyzes GTP with low rates in the absence of Mg^2+^

Involvement of the IM30 protein in membrane protection and/or membrane dynamics in chloroplasts and cyanobacteria has been described in the past, and recently, an apparent GTPase activity of *Ara*IM30 has been observed^39^. Yet, this observation was unexpected, as none of the known IM30 protein sequences does contain predicted domains or amino acid motifs known to be crucial for nucleotide binding and/or hydrolysis (not shown). Thus, the question arose whether the presumed GTPase activity is *Ara*IM30-specific or more generic. Therefore, in the present study, we analyzed the GTPase activity of *Syn*IM30. We here show that *Syn*IM30 can bind GTP, resulting in GTP hydrolysis and release of free P_i_. This apparent GTPase activity can be modulated by a classical GTPase inhibitor (GMP-PCP).

In fact, when analyzed with the malachite green assay, *Syn*IM30 has a (rather low) GTPase activity with a *k*_*cat*_ of 2.16 ± 0.33 min^−1^, a value comparable to the activity described for some other membrane remodeling GTPases, such as dynamin in absence of membranes (*k*_*cat*_ 2.60 ± 0.98 min^−1^)^49^. However, it is worth mentioning that the *k*_*cat*_ determined using the malachite green assay (Fig. 1b) is essentially derived from the activity averaged over the entire incubation time, and the determined values do thus not account for any bias on the turnover rates, such as substrate limitation/substrate inhibition, *etc*. In an attempt to address these limitations, we applied a continuous phosphate release assay (Fig. 1c) that is in principle capable of measuring the actual rate of P_i_ release and can therefore be used to determine the initial, non-biased turnover rate ${v}_{0}$. Unfortunately, when using this assay we were limited to a very small concentration of the substrate (10 µM GTP), which was significantly lower than the *K*_*M*_ value determined for *Syn*IM30 with the malachite green assay (*K*_*M*_ 0.65 ± 0.14 mM). Based on the parameters obtained for the Hill-equation, the saturation level of IM30 at this GTP concentration is about 0.5%. Thus, under these conditions, the expected *k*_*cat*_ is about 0.01 min^−1^, which is reasonably well in agreement with the experimentally observed *k*_*cat*_ (0.0179 ± 0.02 min^−1^) (Fig. 1c). However, we were not able to observe GTP hydrolysis when using a PK/LDH-coupled assay (Fig. 1d) that is well-established in the NTPase field. Assuming the same activity as observed with the malachite green assay, we would expect to observe a change of OD 0.124/30 min, which should be easily detectable. This can either be explained by IM30 inhibiting the assay and/or *vice versa* or by slow *k*_*off*_ rates for GDP.

Nevertheless, *Syn*IM30 clearly shows GTP hydrolysis activity, as demonstrated by the luciferase-coupled GTPase assay, which directly depends on the decrease of the GTP concentration. Again, the observed rate is in reasonable agreement with the *K*_*M*_ and *k*_*cat*_ values obtained from the malachite green-based assay, taking the low GTP concentration of 4 µM into account. However, IM30 binds GTP rather weakly, compared to *K*_*D*_ values of typical GTPase. *E.g*. GTPases of the *ras* superfamily, which are considered high-affinity GTPases, have *K*_*D*_ values in the subnanomolar range^50^, whereas low-affinity GTPases, such as dynamins or the signal recognition particle (SRP), have *K*_*D*_ values in the range of 0.5–5 µM^50^. Thus, typical low-affinity GTPases still bind GTP with at least 100-fold higher affinities than observed here for *Syn*IM30.

Addition of GMP-PCP, a non-hydrolyzable GTP analog, revealed strong cooperative properties of the apparent GTPase activity, which was essentially not observed in the absence of GMP-PCP. Within the frame of an allosteric two-state model, both allosteric and competitive binding could produce such an increase in cooperativity (see Suppl. Fig. 3). While the data did not allow to identify the exact nature of GMP-PCP inhibition, the observations demonstrate that binding of GTP is a cooperative process, probably involving more than four binding sites (see Suppl. Fig. 3d).

While *Syn*IM30 exhibits a GTPase activity, which can be modulated by a non-hydrolyzable GTP analog, this activity clearly is Mg^2+^-independent (Fig. 2a), which is rather uncommon for canonical GTPases. Typically, Mg^2+^ is required for GDP and GTP binding and hydrolysis. Yet, absence of Mg^2+^ does neither affect GTP binding nor GTP hydrolysis by *Syn*IM30 (Fig. 2), and both *K*_*M*_ and ${v}_{max}$ were about identical in presence or absence of Mg^2+^ (Fig. 2a). While binding of GTP and an apparent GTPase activity in absence of Mg^2+^ have already been described for other GTPases, involving FtsZ^51^ or Rho family GTP-binding proteins^52^, in these cases Mg^2+^ addition dramatically increased the hydrolysis rates. In fact, here Mg^2+^ binding was suggested to regulate the kinetics of GTP binding and hydrolysis to achieve high catalytic efficiency and specificity. Thus, a fully Mg^2+^-independent GTP hydrolyzing activity is rather uncommon. Nevertheless, while ${v}_{max}$ remained unchanged in presence of Mg^2+^, the cooperativity of GTP hydrolysis by the oligomeric IM30 was significantly increased. Mg^2+^ is known to directly bind to *Syn*IM30 and to alter the structure of *Syn*IM30 oligomers^47^, which, *vice versa*, *e.g*. results in an increased thermal stability of the protein (Fig. 2b). Thus, a model, where binding of Mg^2+^ leads to a shift in the conformational distribution between different structural states, is reasonable and explains the observed change in cooperativity well, while ${v}_{max}$ remains constant.

### GTP hydrolysis depends on the oligomeric state

While IM30 proteins do not contain any canonical GTPase domain or any motif known to be involved in NTP binding and/or hydrolysis, Ohnishi *et al*. have identified helix 1 of *Ara*IM30 to be crucial for GTP hydrolysis, as deletion of helix 1 resulted in reduced GTP hydrolysis^39^. However, deletion of helix 1 also resulted in disassembly of the typical IM30 high molecular mass complexes in case of the *Ara*IM30 protein^4,39^, and thus the GTP hydrolyzing activity could well be coupled to the oligomeric state of IM30 proteins. In fact, we show here that GTP binding/hydrolysis does depend on the oligomeric state of IM30 but does not crucially involve helix 1. As *Syn*IM30 H2-7 still had considerably high GTP hydrolyzing activity and as we assume a conserved function of the helices in IM30 proteins of different organisms, we rule out the possibility that any amino acid in helix 1 is crucial for GTP binding or hydrolysis. However, the *Ara*IM30 oligomer appears to be less stable than the proteins of *Synechocystis* or *Chlamydomonas reinhardtii*, as in the latter cases helix 1-truncated versions reportedly still form high molecular mass complexes^5,25,46^. Thus, we assumed that oligomer formation, rather than helix 1, is crucial for GTP hydrolysis by IM30 proteins, and we indeed confirmed this assumption by using a *Syn*IM30 full-length mutant protein that forms tetramers but no high molecular mass complexes anymore (Fig. 3b). As this mutant did not hydrolyze GTP anymore, a tetrameric IM30 structure likely is insufficient for GTP hydrolysis and higher oligomeric structures are required, most likely prototypical IM30 rings^7^. As the analyzed *Syn*IM30 mutant forms tetramers, which are considered to be the basic building blocks of IM30 rings^7,37^, the putative GTP binding sites are potentially localized within the ring in between adjacent tetrameric building blocks.

### GTP binding and hydrolysis do not critically affect IM30-mediated membrane remodeling

While we could clearly establish that *Syn*IM30 high molecular mass oligomers do hydrolyze GTP (also in absence of Mg^2+^), the important question arose whether this cryptic GTPase activity has any impact on the physiological function of the protein. Unfortunately, the exact function of IM30 is still not finally resolved. Yet, *in vitro* analyses clearly show that the protein binds to negatively charged membrane surfaces and is able to mediate membrane fusion^16,23,24,37,46^. In fact, in many cases a membrane remodeling activity of NTPases is nucleotide-dependent and typically requires nucleotide hydrolysis (reviewed in^53^). Nevertheless, membrane interaction and membrane remodeling by IM30 clearly do not require nucleotide binding and/or hydrolysis, as NTPs were never present in any assay reported thus far^4,16,17,23,24,37,46^. As a stimulating effect of a lipid surface on a protein’s GTPase activity has been shown for many other proteins, such as dynamin-like proteins^49^, it was well possible that GTP binding and/or hydrolysis modulate the membrane interaction and/or the fusion activity of *Syn*IM30. However, our results suggest that the presence of GTP or GDP does not affect membrane binding of *Syn*IM30, at least in absence of Mg^2+^ (Fig. 4a,b), and also the GTPase activity of *Syn*IM30 is not significantly enhanced in presence of DOPG liposomes (Fig. 1f). However, a minor effect on GTPase activity might have been unnoticed due to the rather low affinity of IM30 to lipid membranes^37^, leaving a considerable fraction of the protein unaffected upon presence of the membranes.

Analysis of the fusion events was challenging, as direct binding of Mg^2+^ is necessary for IM30 activation^23,47^ as well as for membrane destabilization, when Mg^2+^ is interacting with negatively charged liposomes^54–59^. Thus, while the exact same concentration of “free” Mg^2+^ is necessary for comparing the fusion data, the amount of free Mg^2+^ is clearly altered in the presence of GTP or GDP due to the nucleotide-Mg^2+^ interaction^48^. We minimized this effect via experimentally adjusting the concentration of Mg^2+^ specifically when GTP or GDP was present (as described in the “Experimental Procedures” section). Nevertheless, we cannot rule out that the observed differences of the fusion kinetics (Fig. 5) were biased by our approach to experimentally determine the required Mg^2+^ concentrations in presence of GTP or GDP to gain similar fusion curves in absence of IM30 (Suppl. Fig. 4).

Thus, the results of the membrane fusion assay have to be treated with caution. Yet, the kinetics of IM30-mediated membrane fusion were somewhat altered in the presence of GDP or GTP. While GTP enhanced the fusion rate, the membrane fusion rate was somewhat decreased in the presence of GDP (Fig. 5).

However, any influence of potential direct interaction of the nucleotides with the lipid bilayer, as possibly observed in the present study (Fig. 4a and b), is not considered in our analysis. In fact, in presence of Mg^2+^, GTP or GDP could well interact with the negatively charged DOPG head group, as Mg^2+^ can “bridge” the phosphate groups of the nucleotides and the PG head group. This has *e.g*. been discussed for binding of DNA to anionic and zwitterionic membrane surfaces^60,61^. Consequently, nucleotides binding to the membrane surface might directly affect fusion-related membrane properties, which eventually alters fusion kinetics. Furthermore, membrane fusion is affected by multiple, complex equilibria involving IM30, Mg^2+^, liposomes and the nucleotides that are furthermore constantly changing during membrane fusion and GTP hydrolysis. The small changes in the fusion rate observed here are consequently no decisive hint for any influence on the membrane fusion. Importantly, major impacts on the IM30 (*in vitro*) fusion activity can be detected via the fusion assay, as e.g. observed analyzing IM30 mutants^7^.

We conclude that GTP binding and/or hydrolysis are no key regulators of IM30 membrane binding or membrane fusion. If at all, GTP binding/hydrolysis only slightly modulates the kinetics of the membrane fusion process. Hence, membrane remodeling by *Syn*IM30 is vastly nucleotide-independent, despite the cryptic GTP hydrolyzing activity.

### IM30 is an atypical GTPase

Besides apparently hydrolyzing GTP, at least *in vitro*, *Syn*IM30 does not exhibit features expected for a canonical GTPase, involving (i) a G-domain and (ii) Mg^2+^ dependent substrate binding.

Although the G-domain or the P-loop motif, respectively, are fingerprints of GTPases, a few examples of G-proteins lacking the P-loop motif are described^62^. The most extensively studied members of such untypical GTPases are Tubulin and FtsZ, which have a highly conserved nucleotide-binding site that clearly differs from typical GTPases^63^. While IM30 proteins do not contain any sequences involved in nucleotide binding to any thus far studied protein, we cannot *per se* exclude that GTP binds to a new, currently not described GTP-binding pocket to IM30 proteins. Furthermore, as our results suggest that GTP hydrolysis requires oligomeric structures larger than tetramers, it is well possible that amino acids of different IM30 monomers together form the GTPase site, which would not be easily predictable and could only be visualized in a high-resolution structure of oligomeric IM30. Unfortunately, neither a high-resolution structure of an oligomer nor of the monomer is available for IM30 yet. Thus, structural comparisons are limited to homology modeling based on the structure of the PspA coiled-coil fragment that was solved recently^64^. However, no NTP-binding site was identified in such a model^7^.

Furthermore, as outlined above, typically nucleotide binding and hydrolysis depend on Mg^2+^ and in cases where nucleotides are hydrolyzed even in the absence of Mg^2+^, the hydrolysis rates increased in the presence of Mg^2+^. In contrast, here we show that the cooperativity of the apparent IM30 GTPase activity is increased when Mg^2+^ is absent, which could well be ascribed to an allosteric action of Mg^2+^ (see Suppl. Fig. 3). Binding of Mg^2+^ to IM30 has been demonstrated recently, and Mg^2+^ binding clearly induces structural rearrangements of oligomeric IM30^47^, in line with the allosteric function of Mg^2+^. These structural rearrangements appear to affect the GTP hydrolysis activity of oligomeric *Syn*IM30, as discussed above. The GTP hydrolyzing activity of *Syn*IM30 was altered in the presence of GMP-PCP, a non-hydrolyzable GTP analog, typically used as a competitive inhibitor. This lead to a further increase in cooperativity, due to an increased population of a low-affinity state.

Taken together, IM30 exhibits many characteristics that clearly separates it from canonical GTPases, such as the missing G-domain and Mg^2+^-independent GTP hydrolysis. Thus, IM30 might found a new class of GTPases^39^. Yet, GTP binding and hydrolysis clearly have no major effect on the membrane remodeling activity of IM30, at least *in vitro*, and it remains to be shown whether GTP hydrolysis by IM30 proteins is relevant *in vivo*.

## Experimental Procedures

### Cloning, expression and purification of IM30

Construction of the plasmid used for expression of N-terminally His-tagged *Synechocystis* IM30 (pRSET IM30 WT) was described recently^13^. The plasmid used for expression of helix 1 truncated *Syn*IM30 is described in^46^. The plasmid used for expression of the IM30 FERM_EE mutant was created via introducing six mutations (E83A, E84A, F168A, E169A, R170A, M171A) into IM30 WT using the Quick-Change method^65,66^.

All proteins were expressed in *E.coli* BL21 (DE3) at 37 °C for 3–4 h after induction via the addition of 0.5 mM Isopropyl-β-D-thiogalactopyranoside (IPTG).

Cells were resuspended in lysis buffer (50 mM HEPES pH 7.6, 300 mM NaCl, 20 mM imidazole), lysed via sonification (4 °C) and cell debris was removed by centrifugation (8200 g, 10 min, 4 °C). His-tagged IM30 was isolated via Ni^2+^-affinity-chromatography (Ni-NTA agarose matrix, 3 wash steps with 20, 50 and 100 mM imidazole, elution with 1000 mM imidazole). After isolation of the protein, the buffer was exchanged to 20 mM HEPES pH 7.6 by gel filtration (Sephadex G25). Where necessary, the protein solution was concentrated using a Centriprep filter unit (MWCO 10 kDa, regenerated cellulose membrane, 3000 g, 30 min, 4 °C). The protein concentration was estimated using a Bradford assay with a BSA standard curve. For storage, the protein solution was mixed 1:1 (v/v) with glycerol and stored at −20 °C. The purification was completed within 8 hours (time from cell lysis to storage at −20 °C).

### Malachite green assay

*Syn*IM30 (0.1 µM) was mixed with 0.5 mM (if not stated otherwise) of GTP and Mg^2+^ (2.5 mM if not stated otherwise) in 20 mM HEPES and incubated for 30 min at 37 °C. 200 µL of this sample was transferred to a 96-well plate and mixed with 50 µL of the malachite green reaction mixture (“Gold mix”, PiColorLock Gold Phosphate Detection Kit by Innova Biosciences) and incubated for 5 min at RT. Finally, 20 µL of “stabilizer” (PiColorLock Gold Phosphate Detection Kit by Innova Biosciences) was added. Absorbance at 635 nm was measured with an OMEGA FLUOstar Platereader (BMG Labtech). Buffer blank (including the respective GTP concentration) absorbance was subtracted, and the concentration of released phosphate was determined by linear regression from a phosphate standard curve.

The phosphate release value was used to calculate the average NTPase activity *A*_*NTPase*_ [min^−1^] per mol protein:

1 $${A}_{NTPase}=\frac{\Delta [{P}_{i}]}{\Delta t}$$

*[P*_*i*_*]* refers to the concentration of released phosphate and *t* to the incubation time of the hydrolysis reaction.

The Michaelis-Menten constant *K*_*M*_ was determined by using a Hill equation-derived fitting model and the resulting ${V}_{max}$ was used to determine ${k}_{cat}$:

2 $$\begin{array}{ccc}{A}_{NTPase} & = & \frac{{V}_{max}\ast {[S]}^{n}}{{{K}_{M}}^{n}+{[S]}^{n}}\\ {k}_{cat} & = & \frac{{V}_{max}}{[IM30]}\end{array}$$

*[S]* denotes for the substrate concentration and *n* for the Hill coefficient.

For a more detailed analysis of the dependence of GTPase activity on GTP, Mg^2+^ and GMP-PCP, a two-state model was fitted to the data. This is described in more detail in the supplement.

### Continuous phosphate release assay

*Syn*IM30 was mixed with a phosphate sensor (recombinant *E. coli* phosphate-binding protein labeled with the fluorophore MDCC; Sigma Aldrich)^40^ and Mg^2+^ in a 96-well plate. The mixture was preincubated at 37 °C and prewarmed GTP was added directly via an automatic titrator prior to the measurement (OMEGA FLUOstar Platereader (BMG Labtech)). The final concentration in the wells were 0.1 µM protein, 2.5 mM Mg^2+^ and 10 µM GTP. Fluorescence of the samples was measured at 37 °C with a 420/10 nm excitation filter and a 460/10 nm emission filter over the indicated timescale.

From the change in the fluorescence signal over time (after subtraction of the fluorescence signal generated by GTP auto-hydrolysis in the buffer), the concentration of released phosphate was determined via linear regression using a phosphate standard curve. The initial, non-biased turnover rate ${v}_{0}$ [min^−1^] was determined by a monoexponential fit for non-linear, steady-state enzyme kinetics, assuming a first-order process^41^:

3 $$[{P}_{i}](t)=\frac{{v}_{0}}{\eta }\ast (1-{e}^{-\eta \ast t})$$

*[P*_*i*_*]* is the measured concentration of released phosphate and $\eta$ accounts for a term describing the bias on ${v}_{0}$ that causes non-linearity, *e.g*. reduced velocity due to substrate limitation or product inhibition^41^. ${v}_{0}$ was used to determine the ${k}_{cat}$:

4 $${k}_{cat}=\frac{{v}_{0}}{[IM30]}$$

### PK/LDH-coupled GTPase assay

The reaction buffer containing 5 mM MgCl_2_, 150 mM NaCl, 7.5 mM KCl, 0.6 mM NADH, 1 mM PEP (phosphoenolpyruvate), PK(final concentration 12–20 units/mL)/LDH(final concentration 18–28 units/mL) mix (Pyruvate Kinase /Lactic Dehydrogenase mix from Sigma-Aldrich) (and 0–5 mM GTP in 20 mM HEPES pH 7.6 was preincubated at room temperature for 15 min to convert already present GDP to GTP. *Syn*IM30 WT or *Syn*DLP was mixed with the reaction buffer to a final concentration of 0.5 µM. The GTP to GDP turnover rate was determined indirectly via the decrease of NADH absorbance at 340 nm^43^ on a timescale of 1–2 h at 37 °C with an OMEGA FLUOstar Platereader (BMG Labtech). The decline in NADH concentration is proportional to the concentration of GDP produced. The slope of the measured NADH absorbance was determined via fitting with a linear function. The GTPase activity ${A}_{NTPase}$ [min^−1^] was calculated as follows:

5 $${A}_{NTPase}=-\frac{\Delta {A}_{340}}{\Delta t}\ast {l}^{-1}\ast {{\varepsilon }_{NADH}}^{-1}$$

with $\frac{\Delta {A}_{340}}{\Delta t}$ being the linear slope of the absorbance at 340 nm, *l* the filling height of the well and ${\varepsilon }_{NADH}$ the extinction coefficient of NADH (ε = 6220 M^−1^ cm^−1^). Data were corrected for autohydrolysis and the 0 mM GTP value was subtracted from all values to account for protein caused perturbations.

The maximum GTP turnover rate *V*_*max*_ was determined via Eq. 2. The GTP turnover rate was used to determine *k*_*cat*_.

### Luciferase-coupled GTPase assay

To analyze the GTPase activity of IM30 by directly measuring changes in the GTP concentration, we used a luciferase-coupled GTPase assay (GTPase-Glo Assay Kit by Promega). The assay was performed according to the instructions of the manufacturer. In short, 4 µM GTP and IM30 (0–6.5 µM IM30) were incubated for 120 min at 37 °C. After the reaction, the remaining GTP was converted to ATP by addition of GTPase-Glo Reagent (including ADP and a luciferase) and further incubation for 30 min at 37 °C. After 10 min incubation with the detection reagent at 37 °C, the luminescence was measured in a 384-wellplate using an OMEGA FLUOstar Platereader (BMG Labtech). GTP concentrations were calculated from the luminescence intensities using GTP standards that were treated as the samples.

### Size exclusion chromatography (SEC)

Proteins were analyzed on an ÄKTA basic system (GE Healthcare) with a Superose12 10/300 GL column (GE Healthcare) equilibrated with 20 mM HEPES pH 7.6 at 8 °C. Protein elution was monitored via absorbance at 280 nm. The column was calibrated against standards of known size (blue dextran >2000 kDa, β-amylase (200 kDa), alcohol dehydrogenase (150 kDa), albumin (66 kDa), ribonuclease A (13.7 kDa).

### Thermal denaturation

Thermal stability of IM30 (3.2 µM) was determined via CD spectroscopy (JASCO-815 CD spectrometer with an MPTC-490S temperature-controlled cell holder) in presence and absence of 0.5 mM GTP and/or 2.5 mM Mg^2+^. CD spectra were collected from 250 to 200 nm (cell length 0.1 cm, 1 nm data pitch, 5 nm bandwidth, 200 nm/min, 1 s data integration time, averaged over 3 accumulations of the same sample) over a temperature ramp from 15 to 95 °C (2 °C steps, overall heating rate 0.27 °C/min). Spectra were smoothened with a Savitzky-Golay filter. The ellipticity at 222 nm was used as a measure for the folding state of the protein. To estimate the transition temperature *T*_*m*_ of the protein, an indicator for the thermal stability, the resulting melting curve was fitted with an adapted Boltzmann-Fit, which allows linear slopes in the plateau regions of the curve, assuming a simple two-state unfolding mechanism:

6 $${\theta }_{meas}(T)=\frac{(T\ast {m}_{N}+{\theta }_{N})-(T\,\ast \,{m}_{D}+{\theta }_{D})}{1+\,{e}^{\frac{T-{T}_{m}}{dT}}}+(T\ast {m}_{D}+{\theta }_{D})$$

${\theta }_{meas}$ refers to the measured ellipticity. *T* is the temperature, *θ*_*N*_ and *θ*_*D*_ are the ellipticities at the plateaus of native and denatured protein, *m*_*N*_ and *m*_*D*_ are the slopes of the respective plateaus.

### Liposome preparation

The lipids DOPG (1,2-dioleoyl-*sn*-glycero-3-phosphoglycerol), MGDG (monogalactosyldiacylglycerol) and the fluorescently labeled lipids NBD-PE (1,2-distearoyl-*sn*-glycero-3-phosphoethanolamine-N-(7-nitro-2-1,3-benzoxadiazol-4-yl) and LissRhod-PE (Lissamine Rhodamine PE; 1,2-Dioleoyl-*sn*-glycero-3-phosphoethanolamine-N-(lissamine rhodamine B sulfonyl) (ammonium salt)) were purchased from Avanti Polar Lipids, Inc. (Birmingham, AL, USA). For liposome preparation, lipids were dissolved in chloroform/methanol (2:1, v/v). The organic solvent was evaporated under a gentle stream of nitrogen gas followed by overnight vacuum desiccation to remove any traces of solvent. Unilamellar liposomes were prepared by hydration of the dried lipid film with 20 mM HEPES buffer (pH 7.6) and five cycles of freeze-thawing. For the liposome fusion assay, the liposomes were subsequently extruded 15 times through a 100-nm filter, using an extruder from Avanti Polar Lipids, Inc. (Alabaster, AL, USA).

### Laurdan fluorescence measurements

Laurdan (6-dodecanoyl-*N*,*N*-dimethyl-2-naphthylamine) is a fluorescent dye that incorporates into lipid bilayers. Its fluorescence is sensitive to changes in the polarity of the environment and is therefore used to report changes of the membrane fluidity. In order to quantify the spectral changes, the Generalized Polarization (GP) value defined by^67^ is calculated for each spectrum.

7 $$GP=\frac{{I}_{440}-{I}_{490}}{{I}_{440}+{I}_{490}}$$

here, I_440_ and I_490_ are the fluorescence emission intensities at 440 and 490 nm, respectively.

Laurdan (Sigma, Taufkirchen, Germany) was added to the dissolved lipid DOPG in a molar ratio of 1:500. Unilamellar liposomes were prepared as described before. To analyze the effect of nucleotides on the binding of IM30 to DOPG, 1 µM IM30 WT, 0.1 mM liposomes and 2.5 mM GTP (or GDP) were mixed and incubated for 2 h at 25 °C. As the protein was stored in 50% glycerol, the final concentration of glycerol was 15%. For samples without IM30, the corresponding amount of 50% glycerol (20 mM HEPES, pH 7.6) was added.

The fluorescence emission spectra were recorded on a FluoroMax-4 spectrometer (Horiba Scientific, Kyoto, Japan) from 400 to 550 nm with excitation at 350 nm at 25 °C. The slit width was set at 4 nm for excitation and emission of Laurdan.

### Liposome fusion assay

The influence of nucleotides on IM30-triggered liposome fusion was measured using a FRET-based assay, as described earlier^23,68^. Unlabeled liposomes were mixed in 10-fold excess with labeled liposomes containing two fluorescent dyes that form a FRET-pair. Upon fusion of labeled with unlabeled liposomes, the FRET dyes redistribute over the membrane and consequently FRET decreases, resulting in increasing donor emission intensity. To simulate complete membrane fusion, liposomes containing only a 10^th^ of the fluorescently labeled lipids were used as a positive control.

For the fusion assay, 1 µM IM30 WT and 0.1 mM liposomes (MGDG/DOPG, 40:60, w/w) were used. As described for the Laurdan measurements, the final concentration of glycerol was 15% for all samples. The concentration of MgCl_2_ was 6 mM for samples without nucleotides.

The measurements were performed using a Fluoromax-4 spectrometer (Horiba Scientific, Kyoto, Japan). The IM30-containing solutions were preincubated with Mg^2+^ for 15 min, whereas the liposomes were premixed with the nucleotides for about 1 min. After fast mixing of all compounds, the measurement was started immediately. Upon excitation of the FRET-donor NBD-PE at 460 nm, the donor emission was monitored at 525 nm over 1000 s at 25 °C. The slit widths for excitation and emission were set at 5 nm.

The raw fluorescence data was converted to a fusion rate in percentage by Eq. 8 using the intensities of the negative control (*I*_*NC*_), positive control (*I*_*PC*_) and the measured sample (*I*) at every point in time *t*.

8 $$Fusion(t)=\frac{{I}_{t}-{I}_{t,NC}}{{I}_{t,PC}-{I}_{t,NC}}\cdot 100{\rm{ \% }}$$

The initial fusion rate was determined by the first derivative of the fusion curve after 50 s.

Since GTP and GDP bind Mg^2+^ via their phosphate groups, higher concentrations of MgCl_2_ had to be used to obtain the same amount of free Mg^2+^ in samples containing GTP or GDP as in the control. To determine the additionally needed amount of Mg^2+^, we made use of the fact that Mg^2+^ alone can induce fusion at a concentration of 10 mM. Increasing amounts of MgCl_2_ were added to the assay containing 10 mM Mg^2+^ in presence of 2.5 mM GTP or GDP, until the observed fusion rates matched the one obtained in absence of the nucleotides. In the case of GTP, 4.25 mM MgCl_2_ was additionally needed, in the presence of GDP 2.5 mM MgCl_2_ (Suppl. Fig. 2).

## Supplementary information

Supplementary Information.
