# Supplementary material for: GTP hydrolysis by Synechocystis IM30 does not decisively affect its membrane remodeling activity
Source: Sci Rep. 2020 Jun 17;10:9793. doi: 10.1038/s41598-020-66818-9 (PMC7299955; doi:10.1038/s41598-020-66818-9)
Supplement: Supplementary file 1 — Supplementary Information. [file 41598_2020_66818_MOESM1_ESM.pdf]

Title: GTP hydrolysis by *Synechocystis* IM30 does not decisively affect its membrane remodeling activity

Authors: Benedikt Junglas[#], Carmen Siebenaller[#], Lukas Schlösser, Nadja Hellmann and Dirk Schneider*

Department of Chemistry, Biochemistry, Johannes Gutenberg University Mainz, 55128 Mainz, Germany
#These authors contributed equally
*Corresponding author

Email address of Author for contact: Dirk.Schneider@uni-mainz.de

Author for Contact details: Dirk Schneider, Johannes Gutenberg University Mainz, Department of Chemistry, Johann-Joachim-Becher-Weg 30, 55128 Mainz, Germany

Phone: (+49) 6131 39-25833, Fax: (+49) 6131 39-25348, E-mail: Dirk.Schneider@uni-mainz.de

Supplemental Information

Supplemental Data 1

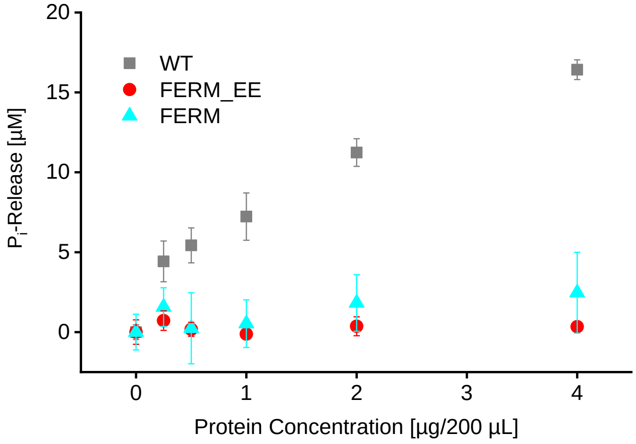

Supplemental Figure 1: **P_i release of IM30 variants**

The P_i release caused by increasing amounts of *Syn*IM30 WT, FERM and FERM_EE in presence of 0.5 mM GTP and 2.5 mM Mg^{2+} during incubation for 30 min at 37 °C was measured using a malachite green-based assay. (SD, n=3).

Supplemental Data 2

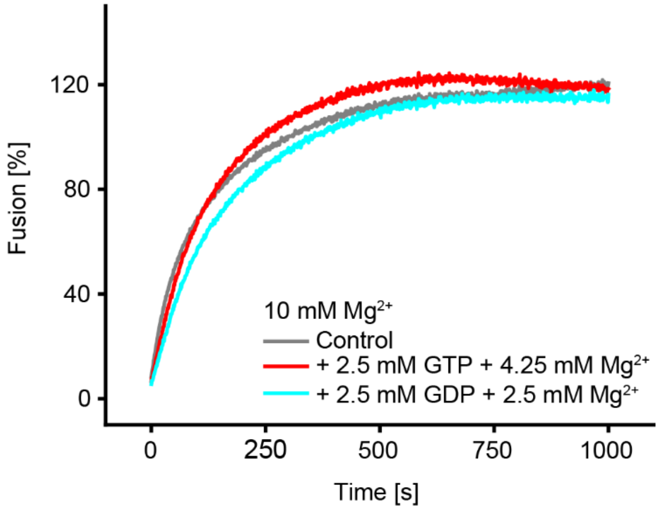

Supplemental Figure 2: **Vesicle fusion rates in the presence of GTP and GDP were adjusted by the addition of Mg^{2+}**

Fusion curves were obtained in the presence of 10 mM Mg^{2+}. In the presence of GTP/GDP, extra Mg^{2+} was necessary to reach the same fusion rates as in the control. (n=3).

Supplemental Data 3

Analysis of the GTPase activity based on a cooperative model

The simplest model taking into account cooperativity in a mechanistic way is the two-state model (aka MWC-model). In this model, an equilibrium between a high-affinity *R* state, and a low-affinity *T* state is assumed, which each is characterized by the corresponding binding constant (or K_M-values) for the substrate (K_R and K_T, respectively). The equilibrium between the two states in absence of substrate is given by the allosteric equilibrium constant *L* and the number of binding sites changing the conformation in a cooperative manner (size of the allosteric unit) is denoted by *n*. The rate of product formation for enzymatic steady state activity is then given by

$$v = v_R \frac{K_R x(1 + K_R x)^{n-1}}{(1 + K_R x)^n + L(1 + K_T x)^n} + v_T \frac{L K_T x(1 + K_T x)^{n-1}}{(1 + K_R x)^n + L(1 + K_T x)^n}$$

With v_R and v_T denoting the v_{max}-values of the *R* and *T* state, respectively.

For numerical reasons, the function fitted to the data was slightly rearranged:

$$v = b_R \frac{x(1+K_R x)^{n-1}}{(1+K_R x)^n + logL(1+K_T x)^n} + b_T \frac{logL\ x(1+K_T x)^{n-1}}{(1+K_R x)^n + logL(1+K_T x)^n} \qquad \text{eq.S1}$$

with $b_x = V_x K_x \ _{x=R,T}$

To each of the three data sets shown in Supplemental Figure 3, eq.S1 was fitted, assuming that the values for v_x, K_x and *n* are the same for all data sets. Assuming that Mg^{2+} acts as an allosteric effector, the two curves determined in absence of GMP-PCP, differing in the Mg^{2+} concentration, were allowed to have different values for *L*. While it might be possible that the values for K_R and K_T differ to some extent due to Mg^{2+}-GTP complex formation, it is not necessary to describe the data. The data obtained in presence of GMP-PCP were analyzed based on two different models: a) GMP-PCP acts allosterically, thus again another value for L was allowed for this data set; b) GMP-PCP acts as a competitive inhibitor, thus logL remains the same as in absence of GMP-PCP. For this case eq. S1 was replaced by

$$v = b_R \frac{x(1 + K_R x + Z_R * 0.5)^{n-1}}{(1 + K_R x + Z_R * 0.5)^n + logL(1 + K_T x + Z_T * 0.5)^n} + b_T \frac{logL\ x(1 + K_T x + Z_T * 0.5)^{n-1}}{(1 + K_R x + Z_R * 0.5)^n + logL(1 + K_R x + Z_T * 0.5)^n}$$

Introducing Z_R and Z_T, which are the binding constants of GMP-PCP to the *R* and the *T* state, respectively. Then, the three corresponding functions were fit simultaneously to the three data sets, rendering convincing

results for both models (Supplemental Figure 3). In case of competitive binding of GMP-PCP, the errors for Z_R and Z_T were very high (Supplemental Figure 4), and the values were similar, therefore in a subversion of the competitive model, we set Z_T=Z_R, which also yielded reasonable results. If the number of cooperative binding sites (n) was also allowed to vary during the fitting routine, a value around 10 with very high errors (nearly 100%) was obtained. Therefore, the fitting was performed for different values of n, and the squared residues divided by the degree of freedom was compared (Supplemental Figure 3). Based on this, a size of the allosteric unit n of about 8 or more is suggested, and the allosteric models tend to yield the best fit.

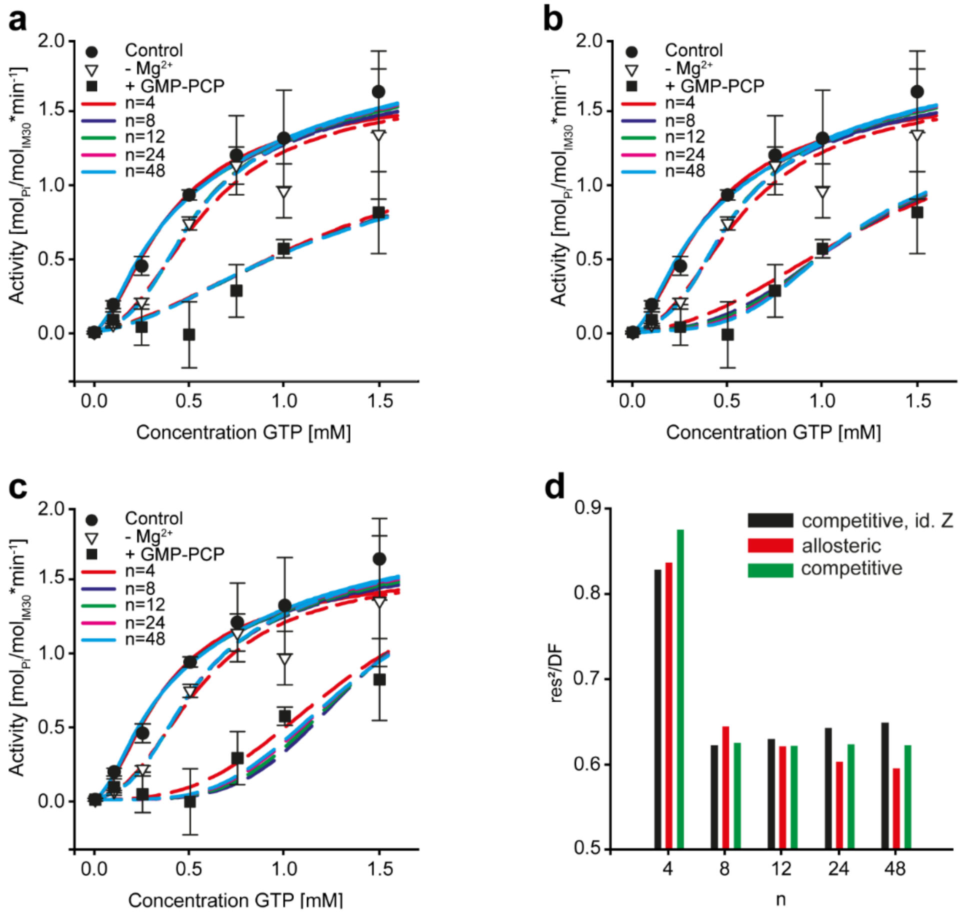

Supplemental Figure 3: **Analysis of GTPase activity based on the two-state model**

The data shown in this figure are replotted from Fig. 3a. Additional fits, based on different size for the allosteric unit n, indicated by different colors and for different models are shown: **a:** competitive model, assuming Z_T=Z_R **b:** competitive model. **c:** allosteric model. Furthermore, the squared residuals (Sq) corrected for the degree of freedoms are compared (**d**), showing the largest difference between n=4 and n=8. Increasing the size of the allosteric unit further slightly reduces Sq in case of the allosteric model, but not for the competitive model.

Furthermore, the values of the parameters obtained for the different values of *n* and models were compared (Supplemental Figure 4).

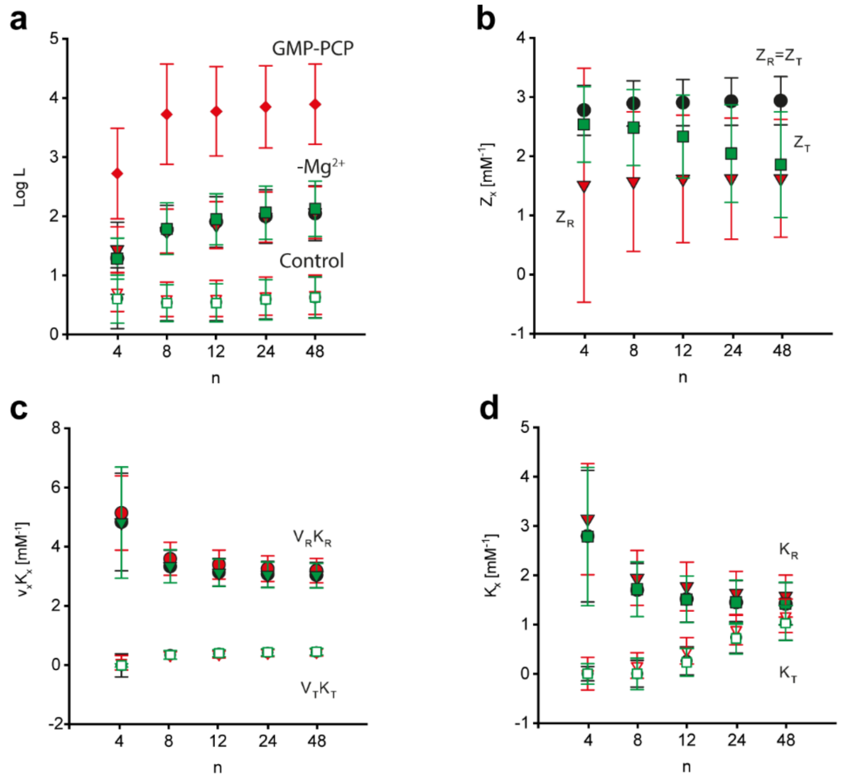

Supplemental Figure 4: **Dependence of model parameters on the size of the allosteric unit**

For each model the data analysis delivered a set of values for K_R, K_T, $b_R=V_R*K_R$, $b_T=V_T*K_T$, logL for the control ("+Mg"), logL for absence of Mg^{2+} ("-Mg") and, in case of the allosteric model, a value for logL in presence of inhibitor ("GMP-PCP"). The results for the different models are presented in different colors: red=allosteric, green=competitive, black=competitive with $Z_R=Z_T$. The error bars were provided by the fitting routine. Since the number of cooperating binding sites (size of the allosteric unit *n*) is not known, the model parameters were determined for different values of *n*. **a:** The allosteric equilibrium constant (logL) increases somewhat with *n*, but clearly differs for the three data sets for n>4 as one would expect if Mg^{2+} and GMP-PCP are allosteric effectors. **b:** The binding constants of GMP-PCP to the R and the T state Z_x do not show a no strong dependence on *n*. **c:** The parameter V_xK_x is independent of n, except at the lowest values tested. **d:** The binding constants K_x decrease with *n*. This is to be expected due to coupling between K_R and logL, which increases with *n* as shown in panel a).

For each model the data analysis delivered a set of values for K_R, K_T, $b_R=V_R*K_R$, $bT=V_T*K_T$, logL for the control ("+Mg"), logL for absence of Mg^{2+} ("-Mg") and, in case of the allosteric model, a value for logL in presence of inhibitor ("GMP-PCP"). Considering the error bars, as provided by the fit routine, the parameters do not depend intensely on the model. Consistently, K_R is larger than K_T, with a decrease in difference with increasing *n*. As expected, logL is very small in case of ("+Mg"), reflecting a curve with nearly no cooperativity, a somewhat increased value of logL in absence of Mg^{2+} and a clear increase in

presence of GMP-PCP. Thus, Mg^{2+} shifts the conformational distribution towards the R-state, while GMP-PCP shifts it effectively to the T-state, in frame of the allosteric model, implying a higher affinity to the T- than to the R-state. In the frame of a competitive model, preferential binding to the T-state is not required to produce an increase of cooperativity (model with $Z_R = Z_T$). The average affinity for GMP-PCP tends to be higher than for GTP in the R-state ($\approx$ 5 mM^{-1} compared to $\approx$ 2 mM^{-1}).
